# Integration of vestibular and visual cues for navigation, visual processing and perception

**DOI:** 10.1146/annurev-neuro-120722-100503

**Published:** 2023-07-10

**Authors:** Sepiedeh Keshavarzi, Mateo Velez-Fort, Troy W. Margrie

**Affiliations:** The Sainsbury Wellcome Centre for Neural Circuits and Behaviour, University College London, 25 Howland Street London

## Abstract

Despite increasing evidence of its involvement in several key functions of the cerebral cortex, the vestibular sense rarely enters our consciousness. Indeed, the extent to which these internal signals are incorporated within cortical sensory representation and how they might be relied upon for sensory-driven decision-making, during, for example, spatial navigation, is yet to be understood. Recent novel experimental approaches in rodents have probed both the physiological and the behavioural significance of self-motion signals and indicate that their its widespread integration with vision improves both the cortical representation and perceptual accuracy of self-motion. We suggest that such vestibulo-visual integration reflects a process of constant updating regarding the status of self-motion, and access to such information by the cortex is used for sensory perception and predictions that may be implemented for rapid, navigation-related decision-making.

## Introduction

Head-movements increase the breath of the sensory world vertebrates survey when engaged in natural behaviour (Ericsson et al. 2012). While these movements are important for perception (e.g., generating motion parallax for depth perception) and spatial orientation (e.g., orienting toward the source of a sound or an odour and planning one’s directional heading), they also pose a challenge for the brain to determine the precise location and motion status of external objects as the observer moves through space. For instance, because motion on the retina can arise either from an external object moving in the scene or from head and eye movements, the brain must disambiguate this retinocentric input by combining it with head and eye motion cues. Beyond compensating sensor movements and maintaining a coherent perception of the external world, the integration of visual and head motion cues also increases the accuracy of self-motion and orientation computations, effectively supporting successful navigation.

The brain can derive head motion information from multiple sources, including vestibular sensors that detect angular and linear acceleration of the head in multiple planes, efference copy of the motor command when movement is self-initiated, and neck muscle proprioception. Electrical stimulation of the vestibular nerve in deeply anaesthetised rodents evokes widespread activation of sensory and motor cortical areas (Rancz et al. 2015) suggesting that self-motion signals are broadcast to brain regions engaged in the self-generation of movement and sensation during locomotion. Over the past few years, studies on primates have significantly advanced our knowledge of how visual and vestibular cues can combine for optimal heading perception (Butler et al. 2010, Fetsch et al. 2011, Fetsch et al. 2009, Gu et al. 2008, Jurgens & Becker 2006, Noel & Angelaki 2022, Prsa et al. 2012). However, the circuit mechanisms that support this multimodal integration, and its functional significance in cortical networks involved in perception and spatial navigation remains poorly understood. Despite differences in the structure and statistics of natural vestibular input between rodents and primates (Carriot et al. 2017), the rodent model presents significant advantages in terms of experimental throughput and the existing array of transgenic and viral tools that can be adapted to study the neural circuits for vestibular processing in general, and vestibulo-visual integration in particular. In addition, while our knowledge of neural computations for navigation is largely based on experiments in rodents, and despite the importance of head motion cues in these computations, experimental data on vestibular signals in the rodent cortex and their integration with visual cues are only just emerging. Here, we summarise and discuss these recent findings, focusing on cortical circuits that are involved in visual perception and spatial navigation. We highlight major unresolved questions regarding the role of vestibular and visual integration during behaviour and the underlying circuit mechanisms, and propose experimental approaches to address them.

## Vestibular and visual integration for navigation

To successfully navigate through the environment, animals rely on their ability to know their directional heading. At least two types of cells identified in the rodent’s brain – as well as in other animals including insects (Green et al. 2017, Seelig & Jayaraman 2015, Turner-Evans et al. 2017), fish (Vinepinsky et al. 2020), birds (Ben-Yishay et al. 2021), bats (Finkelstein et al. 2015) and primates (Baumann & Mattingley 2010, Robertson et al. 1999, Shine et al. 2016) – are thought to subserve the sense of orientation:

1) Head Direction cells (Chen et al. 1994, Cho & Sharp 2001, Taube 1995, Taube et al. 1990a, Taube et al. 1990b), which are tuned, in allocentric coordinates, to orientation of the head in the azimuthal plane, and are thought to form an internal neural compass.
2) Angular Head Velocity cells (Bassett & Taube 2001, Keshavarzi et al. 2022, Sharp et al. 2001, Sharp & Turner-Williams 2005, Spalla et al. 2022), which signal the speed and direction of head turns, and can therefore generate and update the head-direction signal via an angular path integration mechanism (Blair & Sharp 1995, Laurens & Angelaki 2018, McNaughton et al. 1991, Redish et al. 1996, Skaggs et al. 1995, Zhang 1996).

Both of these head orientation signals are primarily vestibular in origin (Keshavarzi et al. 2022, Muir et al. 2009, Shinder & Taube 2011, Stackman & Taube 1997, Valerio & Taube 2016), but they can also be modulated by efference copy of active head movements and neck proprioception (Keshavarzi et al. 2022, Stackman et al. 2003). In addition, the combination of visual input with these signals is important for reliable orientation computations (Arleo et al. 2013, Goodridge & Taube 1995, Keshavarzi et al. 2022, Taube et al. 1990b), as detailed in the following sections.

### Head-direction (HD) cells

The HD signal is thought to be generated by temporal integration of the AHV signal at the interface between the dorsal tegmental nucleus and the lateral mammillary nucleus (Bassett & Taube 2001, Bassett et al. 2007, Blair et al. 1998, Sharp et al. 2001, Taube 2007, Figure 1). It is then relayed via the thalamus to cortical regions of the navigation system where it ultimately integrates with map-like representations in entorhinal and hippocampal networks (Calton et al. 2003, Gerlei et al. 2020, Harland et al. 2017, Peyrache et al. 2017, Winter et al. 2015a). Vision stabilises the HD signal by anchoring it to the external environment (Goodridge & Taube 1995, Taube et al. 1990b). In particular, prominent and stable visual cues known as visual landmarks strongly control the preferred firing direction of HD cells. In the absence of vision, the HD signal drifts over time due to the accumulation of path integration error (Bjerknes et al. 2015, Goodridge et al. 1998), which may explain why our sense of orientation is lost in the dark, especially over long distances. The integration of visual information into the head direction system occurs at the earliest stage of this hierarchical network, with HD cells in the lateral mammillary nucleus already showing landmark control (Yoder et al. 2015, Figure 1).

How are visual landmarks detected, stored and integrated into the HD system? Lesion experiments have highlighted the significance of two cortical regions for transferring landmark information into the HD cell network, postsubiculum (Goodridge & Taube 1997, Yoder et al. 2015) and the retrosplenial cortex (Clark et al. 2010, Figure 1). Lesioning either region leads to impaired landmark control in upstream parts of the HD system. Yet, whether they actively detect and store landmark information by assessing the input from visual cortical areas, or whether they inherit and further integrate it in the HD system, remains unknown. In addition, it is unclear how visual information flows between these areas and whether they provide redundant information or have complementary integrative roles that support HD signalling. Reciprocal connections between retrosplenial cortex and postsubiculum (Sugar et al. 2011, Wyss & Van Groen 1992) and their anatomical proximity further complicates the interpretation of lesion experiments. For instance, it is likely that the observed impact of postsubicular lesions is, at least in part, due to lesions extending into the overlaying retrosplenial and visual cortices (Yoder et al. 2015), or due to the disruption of HD and landmark computations in the retrosplenial cortex following the loss of postsubicular input. Refined circuit-selective inactivation with the use of novel genetic and viral tools (Huang & Zeng 2013) will help disambiguate the specific role that these regions play in detection, storage and integration of visual landmarks into the HD system.

Recent neurophysiological experiments have identified the neural representation of visual landmarks in the dysgranular division of the retrosplenial cortex (Fischer et al. 2020, Jacob et al. 2017, Sit & Goard 2022). In particular, a subset of directional cells in this area are dominated by visual landmarks, showing bidirectional firing in a visually symmetrical two-compartment environment (Jacob et al. 2017, Figure 1). Theoretical work has proposed that these cells assess the stability of visual landmarks and incorporate them into the HD system through Hebbian plasticity between visual and vestibular-dependent HD input (Page & Jeffery 2018). Although experimental evidence in support of this model is yet to be found, it aligns with human neuroimaging data that suggest a role for the retrosplenial cortex in the processing of landmark permanence (Auger & Maguire 2013, Auger et al. 2012). Whether neural representation of landmarks also exists in postsubiculum and how it relates to those in the retrosplenial cortex remains to be determined.

Landmark-dominated directional cells have also been reported in the postrhinal cortex (LaChance et al. 2022, Figure 1), yet lesion experiments suggest that this area does not play a major role in landmark control of thalamic HD cells (Peck & Taube 2017). Nevertheless, considering the substantial reciprocal connections of the postrhinal area with the retrosplenial cortex and postsubiculum (Agster & Burwell 2013), this region may also contribute to visual landmark processing in cortical and hippocampal navigation networks. In addition to revealing the circuit logic of visual landmark integration into the HD network, future experiments should aim to understand how different cortical areas evaluate the stability and spatial location of visual cues to determine whether they can be used as reliable landmarks and the cellular and synaptic mechanisms for integration of landmark information into the vestibular-dependent HD signal.

What is the behavioural significance of the HD signal and its integration with visual cues? Navigation tasks aimed at answering this question have shown mixed results. While in some tasks, no correlation between HD cell activity and navigation performance were found (Dudchenko & Taube 1997, Golob et al. 2001, Muir & Taube 2004), a few studies that employed a homing task – in which the animal has to find its way back to a home base to consume the food it has collected – have shown significant correlation between the stability of the HD signal and heading accuracy during return (Butler et al. 2017, Valerio & Taube 2012, van der Meer et al. 2010, Figure 2a). Moreover, since the preferred orientation of HD cells can drift in the absence of stabilising visual landmarks (Goodridge & Taube 1997, Yoder et al. 2011, Figure 2a), the animal’s heading performance may be impacted in the dark.

However, experimental designs that aim to remove all potential sources of orienting landmark information, such as tactile, sound, and olfactory cues, are required to test the behavioural significance of visual cues during such tasks.

### Angular head velocity (AHV) cells

Visual inputs also influence the AHV system. A recent study identified AHV-tuned cells in the mouse retrosplenial cortex during open field exploration, and subsequently recorded their activity in a head-fixed apparatus that permits isolation of both vestibular and visual contributions (Keshavarzi et al. 2022). These experiments showed that while AHV cells rely on vestibular input, the combination of visual and vestibular cues increases the gain and signal-to-noise-ratio of their tuning function, thus improving the accuracy of encoding the direction and speed of head turns. The contribution of vision to AHV computation may arise from the combined effect of increased luminance (i.e., more spikes; Bouvier et al. 2020), optic flow (more velocity information) and the increased gain of compensatory eye movements (Stahl 2004). Future work should explore these possibilities by manipulating the properties of the surrounding visual stimuli during self-motion. Regardless of how vision improves AHV coding, these findings suggest that the integration of head motion and visual cues can impact the sense of orientation in two ways: 1) it stabilises the HD signal by anchoring it to reliable visual landmarks in the environment, and 2) it optimises the head velocity estimates, which are used to update the HD signal via path integration.

What could be the behavioural significance of the cortical AHV signal? A recent study on escape behaviour in mice has identified a class of cells that encode head orientation relative to a shelter in both the retrosplenial cortex and the superior colliculus (Campagner et al. 2022). Interestingly, the input from the retrosplenial cortex is not only necessary for this shelter-direction tuning in the superior colliculus, but also for accurate and rapid execution of head turns toward the optimal shelter trajectory (Campagner et al. 2022). Reminiscent of egocentric goal-direction cells found in the hippocampus (Sarel et al. 2017), the shelter-direction cells likely rely on continuous updating from AHV cells for rapid navigation-related decisions and behaviours. Whether the retrosplenial projections to the superior colliculus convey the AHV input for further computation of these orientation signals or whether they simply pass on the shelter-direction signal constructed in the cortex remains to be determined, for instance by imaging the activity of collicular-projecting retrosplenial cells during escape. Further experiments that carefully control and manipulate visual cues and test the animal’s orientation ability in more complex and longer paths, which leads to the accumulation of path integration errors, are necessary to fully understand the relevance of visual information in such behaviour.

Beyond their role in generating and updating orientation signals, AHV-tuned cells may be important for perception of self-motion. Perhaps the most exciting line of vestibular research to emerge in the last decade is the demonstration that – similar to humans – rodents can be trained to use their vestibular system to report self-motion and their passive rotation speed in go/no-go rotation discrimination tasks (Velez-Fort et al. 2018; Keshavarzi et al. 2022, Figure 2b). Importantly, mice improve their perceptual accuracy in this task when visual cues are present, and this improvement requires the integration of visual and vestibular inputs (Keshavarzi et al. 2022, Figure 2b). Where in the brain this integration might take place is yet to be determined. Since the activity of AHV cells in the retrosplenial area mirrors these perceptual data, it is plausible that they contribute to self-motion perception. AHV-tuned cells have also been reported in other parts of the navigation system (Hennestad et al. 2021, Spalla et al. 2022) as well as in primary sensory and motor areas (Hennestad et al. 2021, Long et al. 2022). Whether the same or different AHV populations are involved in orientation computations and perception of self-motion, and whether their role differs between brain areas depending on their input-output connectivity, remains to be determined in future work.

**Figure 1:**
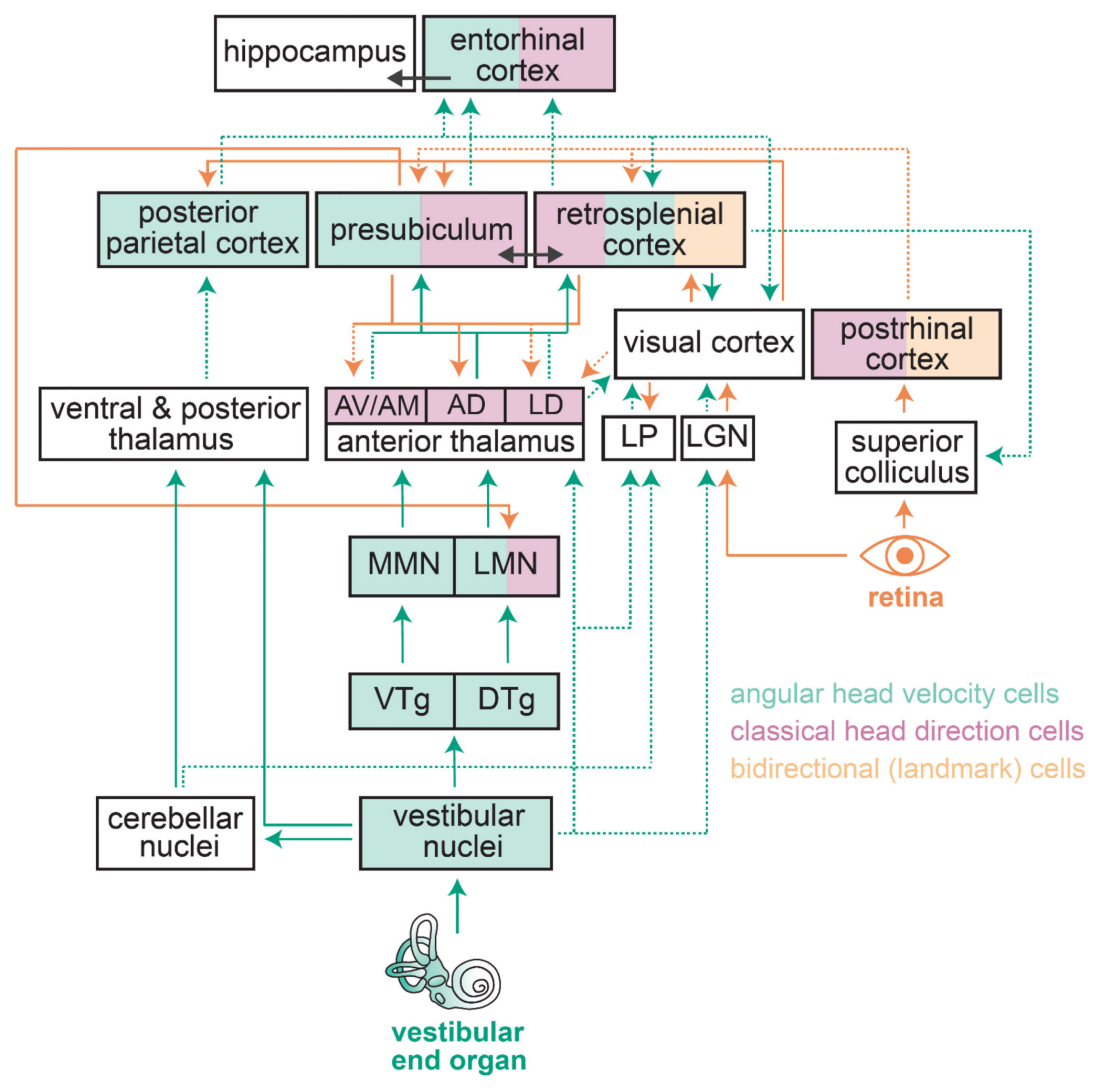
Neural circuits for integration of visual and head motion cues in the rodent brain. Simplified diagram of neural pathways that convey vestibular (green arrows) and visual information (orange arrows) to cortical areas involved in spatial navigation and visual perception. Solid and dashed lines represent established pathways and putative routes that require further experimental evidence, respectively. Arrows show information flow rather than a detailed anatomical connectivity. For simplicity and due to limited data on translational vestibular signals, only angular head velocity and head orientation pathways are shown. AD, anterodorsal thalamic nucleus; AM, anteromedial thalamic nucleus; AV, anteroventral thalamic nucleus; DTg, dorsal tegmental nucleus of Gudden; LD, lateral dorsal thalamic nucleus; LGN, lateral geniculate nucleus; LMN, lateral mammillary nuclei; LP, lateral posterior thalamic nucleus; MMN, medial mammillary nuclei; VTg, ventral tegmental nucleus of Gudden.

Another outstanding question concerns the neural pathways by which the AHV signal reaches the brain’s navigation system (Figure 1). One route may involve the ascending HD system, via projections from the anterodorsal thalamic nucleus to the retrosplenial cortex. However, although this thalamic area contains a large number of HD cells, they are not significantly modulated by AHV (Bassett et al. 2007). Despite this, recent computational modelling data suggest that the AHV signal in the retrosplenial cortex can arise from HD-tuned thalamic afferents as a result of their depressing synaptic dynamics (Brennan et al. 2021). The AHV signal can then reach the other parts of the navigation system either via the retrosplenial cortex (Sugar et al. 2011, Velez-Fort et al. 2018, Wyss & Van Groen 1992) or via other thalamocortical routes, such as direct projections from the anterior thalamus to hippocampal formation and entorhinal cortex (Jankowski et al. 2013). Other vestibulo-thalamic pathways that connect the vestibular and cerebellar nuclei to posterior and lateral thalamic regions (Bohne et al. 2019, Nagata 1986, Shiroyama et al. 1999) may also contribute to the cortical AHV signal. Future recordings and targeted manipulation of activity in these thalamocortical projections can elucidate the neural circuits that convey head-motion information to cortical and hippocampal centres of the navigation system, and further reveal whether the integration of head-motion and visual cues occurs primarily in higher-order cortical networks or is inherited from subcortical regions.

### Translational heading signals

While rotational head-motion inputs contribute to the formation and updating of the neural compass, much less is known about how translational head movements may support navigation. Cells that encode the animal’s linear locomotion speed have been identified in key parts of the navigation system including the medial entorhinal cortex (Kropff et al. 2015, Sargolini et al. 2006), parietal and retrosplenial cortices (Alexander et al. 2022, Cho & Sharp 2001, Keshavarzi et al. 2022, Whitlock et al. 2012) and the hippocampus (McFarland et al. 1975, McNaughton et al. 1983, Wiener et al. 1989). This speed signal is thought to be important for spatial localisation through a path integration mechanism and has been implemented in the vast majority of computational models of spatial coding (Bush & Burgess 2014, Giocomo et al. 2011, McNaughton et al. 2006). The origin of the cortical speed signal is not well understood. Recent data suggest that in the medial entorhinal cortex and hippocampus, it is primarily motor in origin (Winter et al. 2015b) and may be driven by subcortical circuits between the brainstem and basal forebrain (Carvalho et al. 2020, Fuhrmann et al. 2015, Justus et al. 2017) – but also see (Dannenberg et al. 2019, Hinman et al. 2016). Yet, during real-world navigation in freely moving animals, both translational head motion input from the vestibular (otolith) system (Jacob et al. 2014) and optic flow (Chen et al. 2016, Dannenberg et al. 2020, Perez-Escobar et al. 2016) may provide additional information about the animal’s locomotion speed. The respective role of these various motor and sensory cues in generation of the speed signal, how their integration may shape and modulate it, and the neural circuits that propagate them to different cortical regions remain to be determined.

## Vestibular and visual integration for cortical visual processing

Most of our understanding of vestibulo-visual integration in the cortex comes from research on humans and non-human primates. These studies have focused on areas dedicated to visual motion perception, particularly the dorsal medial superior temporal area and the ventral intraparietal area (Thier & Erickson 1992, Duffy 1998; Bremmer et at. 1999, Fasold et al. 2002; for reviews, see Lopez et al. 2011, DeAngelis & Angelaki 2012). Indeed, only a subset of V1 neurons are highly selective for the direction of visual motion and these cells have small spatiotemporal receptive fields that encode visual motion of local features (Hubel & Wiesel 1968). In addition, V1 has a high percentage of neurons suppressed by binocular optic flow (Rasmussen et al. 2021). For these reasons, V1 has not been regarded as well suited for processing self-motion information (Noel & Angelaki 2022).

Nonetheless, indirect evidence supporting the presence of vestibular signals in V1 of humans (Tiecks et al. 1996, Wenzel et al. 1996, Bense et al. 2001) and non-human animal models has existed for decades. For instance, extracellular recordings of neuronal activity obtained in cats during labyrinthine polarization and/or calorization (Grusser et al. 1959, Grusser & Grusser-Cornehls 1960, Jung et al. 1963, Gorgiladze & Smirnov 1967, Grusser & Grusser-Cornehls 1972) or more physiologically-relevant whole-body rotations (Spiegel et al. 1968, Vanni-Mercier & Magnin 1982), have shown a significant proportion of V1 neurons responding to vestibular activation. Comprehensive activation maps of brain regions that respond to vestibular nerve stimulation in rats (Best et al. 2014, Rancz et al. 2015) have now confirmed the presence of cortical vestibular signals not only in V1, but also in other primary sensory cortical areas. Recordings in head-fixed mice from V1 layer 6 in darkness show that many neurons are differentially tuned to the velocity of horizontal rotation (Velez-Fort et al., 2018). Such studies indicate that vestibular modulation of V1 can vary according to cortical depth (Velez-Fort et al. 2018, Bouvier et al. 2020). In superficial layers (layer 2/3 and layer 4), whole-body rotation leads to an overall suppression of neuronal activity, whereas neurons in deep layers (layer 5 and layer 6) are equally distributed between those that are excited and those that are suppressed (Bouvier et al. 2020, but see Velez-Fort et al. 2018). Interestingly, the impact of head motion on V1 activity is light-dependent, since the majority of those units that are suppressed in the dark become excited under ambient light conditions. This light-dependent vestibular response appears to be mediated, at least in part, by a subclass of V1 interneurons (Somatostatin-positive cells in deep layers) that integrate vestibular and luminance signals (Bouvier et al. 2020). Together, these findings demonstrate that head-motion processing and vestibulo-visual integration in V1 occurs in a layer- and cell-type-specific manner.

While evidence for their combined representation at the cortical network level is emerging, the synaptic mechanisms of vestibular and visual integration remains much less studied. *In vivo* whole-cell recordings in V1 show that horizontal rotation can induce both excitatory and inhibitory subthreshold responses that can be direction selective (Velez-Fort et al. 2018). In layer 6, the amplitude of these subthreshold responses is higher when a static visual cue is present compared to rotation in darkness and matches the arithmetic sum of vestibular-only (rotation of the animal in the dark) and visual-only (rotation of the visual cue) responses. While these data suggest that vestibular and visual inputs integrate at the level of individual V1 cells (Velez-Fort et al. 2018), the synaptic mechanisms of this cue integration and how it controls the firing output of individual neurons remain unknown. These questions have been explored to a greater extent outside of the cerebral cortex. For example, in cerebellar granule cells, vestibular processing over a broad range of angular velocities is extremely reliable at the level of individual connections and not prone to short-term changes in synaptic dynamics (Arenz et al. 2008). These cerebellar granule cells also receive visual inputs which can be readily distinguished from the vestibular inputs due to their distinct synaptic properties (Chabrol et al. 2015). Interestingly, when vestibular and visual afferents are simultaneously activated, their integration produces different firing patterns depending on the type of vestibular afferent involved. The representation of combined vestibular and visual sensory input therefore not only causes changes in the firing rate but can also result in discrete changes in the firing pattern of individual neurons. It remains to be determined whether similar principles govern how visual cortical cells integrate these two types of sensory inputs.

One drawback of head-fixed experiments that involve passive rotation or translation is the absence of active head movements. The efference copy of motor commands that initiate head movements in freely moving animals is known to integrate at the earliest stage of central vestibular processing in the vestibular nuclei (Cullen & Taube 2017). Vestibular-only (VO) cells in these nuclei, which are sensitive to vestibular stimulation but insensitive to eye movements, are thought to be involved in maintaining posture, self-motion perception and spatial navigation (Cullen 2019). In mice, these VO cells respond to both passive whole-body rotation and neck proprioception. However, their activity appears to attenuate during active head movements (Medrea et al. 2013), even though, unlike in primates, they still robustly encode head-on-body position during both passive and active motion. Therefore, to fully elucidate the impact of head motion on vision and the role of vestibular signals, it is necessary to investigate these processes in freely-moving animals.

Recent development of head-mounted devices, which allows tracking of eye and head movements while recording neuronal activity, has enabled the study of visual processing in freely moving animals (Voigts et al. 2013; Dugue et al. 2017; Zong et al. 2017; Meyer et al. 2018, Michaiel et al. 2020, Klioutchnikov et al. 2020 & 2022; Parker et al. 2022a, Sattler & Wehr 2020, Wallace et al. 2013; for a review, see Chaplin & Margrie, 2020). With the use of such technical advances, it has been recently discovered that isolated head-movements recorded in the dark in freely moving rodents can modulate the activity of over 50% of V1 neurons (Meyer et al. 2018, Bouvier et al. 2020), and that these head-motion-related responses become increasingly excitatory under light conditions (Bouvier et al. 2020, Guitchounts et al., 2020). Surprisingly, while general movement such as running increases visual responses in V1 (Niell & Stryker 2010), head orienting movements appear to suppress visual-evoked activity (Guitchounts et al. 2020), akin to the attenuation of self-generated sound in the auditory cortex (Rummell et al. 2016, Schneider et al. 2014). Whether such findings reflect differences in integration of linear and angular head motion with vision requires further investigation, but regardless, they confirm that head-motion signals play a major role at the very first stage of cortical visual processing in rodents.

### Vestibular routes into the primary visual cortex

The neuronal pathways that allow V1 to draw on vestibular information are largely unknown, but several candidates, some of which bypass the canonical anterior and ventral/posterior vestibulo-thalamic pathways, have been put forward. Anatomical (Bohne et al. 2019, Magnin & Kennedy 1979, Nagata 1986, Shiroyama et al. 1999) and functional (Papaioannou 1973, Magnin et al. 1974, Magnin & Putkonen 1978; Matsuo et al. 1994) studies in cats and rats have shown potential pathways linking vestibular nuclei to visual thalamic areas such as the lateral geniculate and the lateral dorsal nuclei (Figure 1). In addition, vestibular responses have been reported in the lateral posterior thalamic nucleus (or Pulvinar) in cats and monkeys (Matsuo et al. 1994; Meng et al. 2007; Marlinski & McCrea 2008), and more recently, also in mice (Bouvier et al. 2022 FENS conference abstract). Therefore, multiple thalamic nuclei could serve as the gateway of vestibular information to V1 (Figure 1).

V1 has also been shown to receive angular head velocity information directly from the retrosplenial cortex (Figure 1). Single-cell (Rancz et al. 2011) and population connectivity studies using retrograde rabies tracing have shown that layer 6 cortico-thalamic (CT) neurons in V1 receive significantly more inputs from the retrosplenial cortex, compared to their neighbouring layer 6 cortico-cortical (CC) neurons (Velez-Fort et al. 2014) or layer 2/3 principal cells (Brown et al. 2021). Using a combination of viral tracing tools and calcium imaging, it has been recently discovered that the retrosplenial cortical neurons, which directly synapse onto layer 6 CT cells, respond to whole-body rotation in darkness. These data strongly suggest that at least a fraction of vestibular input to V1 is conveyed via the retrosplenial cortex (Figure 1). It is worth noting that deep layer neurons of higher visual areas also receive a significant fraction of their input from the retrosplenial cortex (Galloni et al. 2022), indicating that the influence of this area on V1 activity might take additional cortical routes. Finally, lesioning large parts of the secondary motor cortex leads to a reduction of head-motion-related responses in V1 of freely moving rats (Guitchounts et al. 2020). The secondary motor cortex is known to receive vestibular input in rodents (Rancz et al. 2015), but the nature of signals inherited by V1 from this area (motor and/or vestibular) remains unknown. Irrespective of whether motor inputs contribute to such head-motion signals, partial lesions of the peripheral vestibular organ almost completely abolish rotation-evoked responses in V1 (Velez-Fort et al. 2018, Bouvier et al. 2020) highlighting their involvement in cortical visual processing.

### Functional relevance of vestibular signals for cortical visual processing

One of the first proposed roles of cortical vestibulo-visual integration is to provide visual cortex neurons with a gravity-based reference frame, which would theoretically preserve visual tuning properties independent of the position of the head. One of the first descriptions of head-angle invariant cells was made in the cat V1 (Horn & Hill, 1969), whereby the receptive fields of a fraction of V1 neurons compensated for body rotation. These studies and others (Horn et al. 1972; Denney & Adornaji, 1972; Metzler & Spinelli, 1979; Tomko et al. 1981) proposed that vestibular, but also proprioceptive information, is integrated in V1 to provide an estimate of the direction of gravity in relation to the visual world. Although only 27% of cells in the cat V1 showed head-angle invariant properties (Tomko et al. 1981), it is the first indication that vestibular and proprioceptive inputs could enable the computation of object orientation independent of head position. In rodents, movement-invariant orientation selective neurons in deep layers of V1 have been described in freely moving rats (Roe et al. 2018 SfN abstract), indicating that vestibular and other types of self-motion inputs can maintain visual tuning properties during natural behaviour. In addition, in freely moving mice, a large fraction of visually-responsive V1 neurons with eye-position and head-orientation tuning have been found (Parker et al. 2022a). In most of these neurons, visual and positional signals combine through a multiplicative (rather than additive) interaction, consistent with a gain field computation (Salinas & Sejnowski 2001), which has been shown to serve coordinate transformations. This could endow V1 with the computational machinery for embedding visual representations within an egocentric reference frame (Parker et al. 2022a).

**Figure 2:**
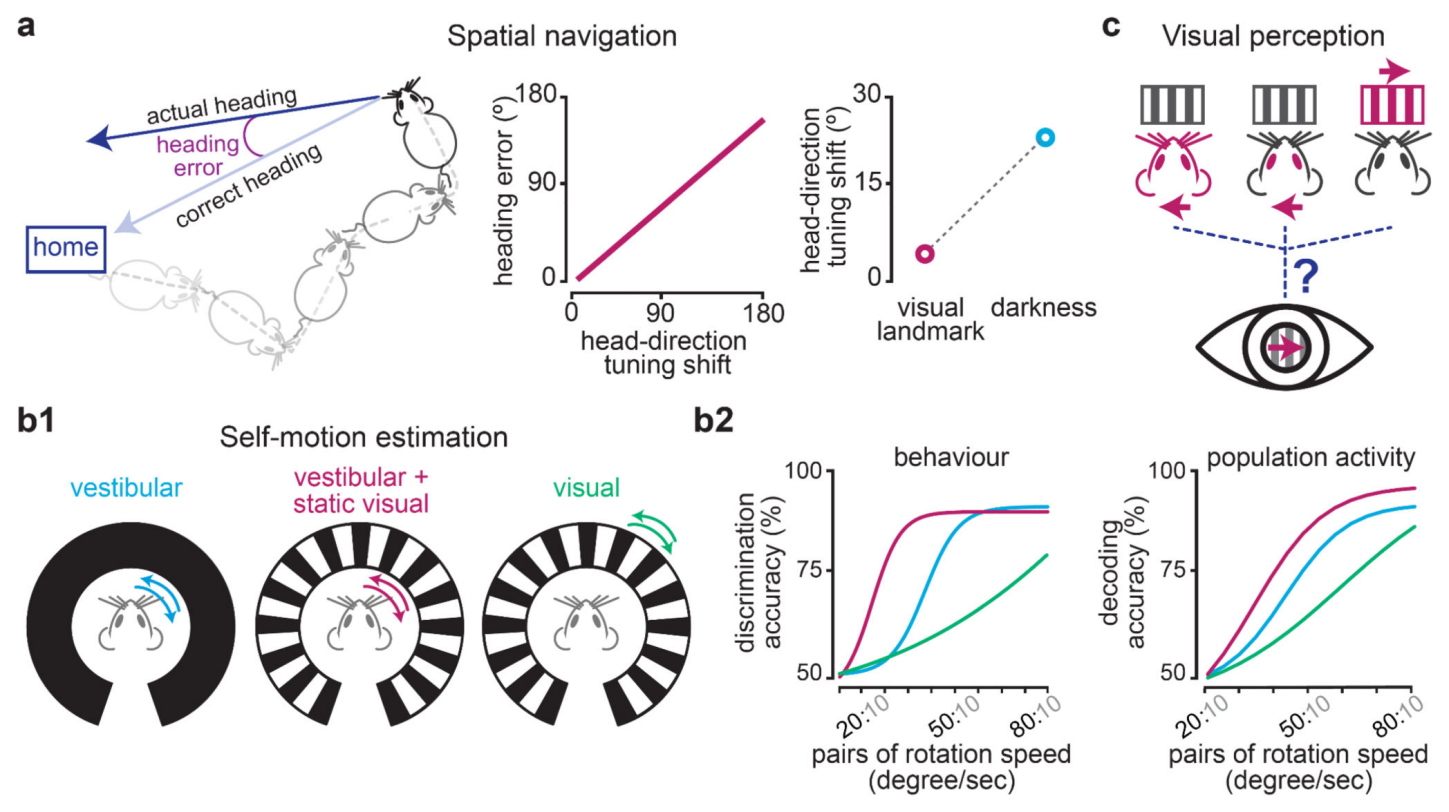
Behavioural significance of the integration of vestibular and visual cues. **a**, The integration of visual cues into the vestibular-dependent head direction signal is important for accurate spatial orientation. The stability of the head direction signal – measured as the amount of shift in the tuning of head-direction cells – is correlated with the rat’s performance in a homing task (left and middle). In the absence of visual cues, the head direction signal becomes less stable (right, increased shift in the tuning of head-direction cells), which may lead to more heading errors. Plots are simplified and based on Valerio & Taube 2012 (left) and Goodridge et al. 1998 (right). **b1-2**, The combination of vestibular and visual cues improves estimation of self-motion. In a rotation-discrimination task in which mice were trained to report their angular speed under different experimental conditions (**b1**), their performance improved significantly when both vestibular and visual stimuli were available (**b2** left, magenta) compared to when rotated in the dark (blue). Similarly, in the retrosplenial cortex, the accuracy of decoding angular self-velocity from population activity increased when both vestibular and visual cues were available (**b2** right) The improved perceptual and decoding accuracy under the multisensory condition could not be explained by the use of vision alone, since both were substantially lower when only visual motion stimuli were present (green). Schematics and plots are simplified and based on Keshavarzi et al. 2022. **c**. The integration of head and visual motion signals is essential for disambiguating the source of motion on the retina, which may arise from head and/or eye movements, or motion in the external world.

Another functional importance of vestibular signals for vision relates to their role in differentiating visual motion generated in the outside world from that caused by the animal’s own movements (Figure 2c). Both the corollary discharge of head- and eye-movement commands (Crapse & Sommer 2008, von Holst & Mittelstaedt 1950) and the vestibular and proprioceptive signals can contribute to this computation. In primates, multiple visual cortical areas contain both cells that have matched vestibular and visual heading preference (‘congruent’ cells) and those with opposing directional tuning for vestibular and visual cues (‘opposite’ cells; Chen et al. 2011, Fetsch et al. 2007 & 2011, Gu et al. 2008). While the activity of congruent cells is consistent with vestibular-visual integration for optimal heading perception and correlates well with perceptual performance, the opposite cells are ill-suited for cue integration (Fetsch et al. 2011, Gu et al. 2008). Instead, these cells respond to a moving object that is not aligned with self-motion and are thought to help dissociate self and object motion. Indeed, simulations (Kim et al. 2016) and neural decoding data (Sasaki et al. 2017) suggest that the combined activity of congruent and opposite cells can resolve the retinal image into components related to head and object motion. Consistent with these data, behavioural experiments suggest that vestibular cues reduce errors in perception of object direction during self-motion (Dokka et al. 2015b) and can eliminate heading biases caused by a moving object (Dokka et al. 2015a). Whether similar computations parse retinal image motion in the rodent’s visual cortex and the extent to which different cortical areas contribute to such computations is yet to be determined.

Vestibular signals in V1 may also contribute to the prediction of visual flow, as previously proposed for motor (stationary running) and coupled optic flow signals (Keller et al. 2012, Leinweber et al. 2017) in a predictive coding framework of cortical function (Keller & Mrsic-Flogel 2018, Rao & Ballard 1999, Spratling 2010). In this framework, vestibular signals are among many types of contextual information – others including the animal’s spatial location and orientation (Guitchounts et al. 2022, Fiser et al. 2016, Saleem et al. 2018, Zong et al. 2022) and self-generated movements (Keller et al. 2012, Leinweber et al. 2017) – that is conveyed to V1 via top-down projections to provide predictions of the bottom-up sensory inputs. The difference between the top-down prediction and bottom-up sensory input is then encoded as a prediction error and passed on to other areas to update the internal representation. Based on this model, when an object is moving in the scene, the animal’s head movement will be accompanied by a bottom-up visual input that is inconsistent with the predicted optic flow, leading to a prediction error that signals external motion. To date, such speculations about the role of cortical vestibular signals have not been directly tested, thus whether vestibular inputs in the visual cortex support the predictive coding model remains an open question (Klingner et al. 2016).

### Uncoupling eye- and head-movement cues

Finally, one of the challenges in studying central vestibular integration is that head and eye movements can be interrelated. It is well known that eye movements modulate the activity of V1 in cats (Toyama et al. 1984) and mice (Meyer et al. 2018; Miura et al. 2022; Parker et al. 2022b), and head movements in freely moving rodents are often accompanied by eye movements (Wallace et al. 2013; Meyer et al. 2018). Several experimental approaches have been undertaken to reduce head-rotation-related eye movements and, in doing so, isolate the contribution of head movements to brain activity. First, eye movements associated with the vestibulo-ocular reflex can be significantly suppressed by pairing the rotation of the head with the rotation of a visual cue (King et al. 1976). This approach, while suitable in head-fixed preparations, is not practical in freely moving animals. Second, eye movements can be abolished or significantly reduced by peri-ocular injections of a local anaesthetic (Velez-Fort et al. 2018) or by eye muscle resection (Bouvier et al. 2020), both of which remove eye movement-related proprioceptive cues. While these surgical approaches have confirmed that at least during passive rotation in mice, head-motion-evoked activity of V1 neurons is primarily vestibular in origin, the effect of the occlusion of eye movements in freely moving animals is unknown. Recording the activity of V1 neurons in freely moving animals that can't move their eyes could provide important novel insights into the independent role of head movements in central visual processing.

## Concluding remarks

Over the past decade or so, there has been considerable emerging evidence that the vestibular modality is pervasive with regard to both its anatomical distribution throughout the cortex and its role in integrative computations. Recent data on vestibulo-visual combination in various cortical networks have now opened the door to study its functional role during behaviour. In particular, the use of rodent models which allow high-throughput neuronal recording and precise circuit dissection has provided novel insights into the neural underpinnings of this multisensory integration for visual processing and spatial navigation. We have outlined these recent findings and identified the knowledge gaps that require further research, with some of the key open questions summarised below. Addressing these shortcomings in the coming years will not only help us better understand the principles of cortical operations, but will also advance our knowledge of neuronal computations underlying perception and naturalistic behaviours.

Unresolved issues

1- What are the routes by which vestibular information reaches the cortex? What are the routes by which optic flow and visual landmark information reaches the head-direction network?
2- Does the integration of vestibular and visual cues occur de novo in the cortex or is the integrated signal largely inherited from subcortical regions?
3- What are the synaptic mechanisms of vestibulo-visual integration in the cortex?
4- What is the behavioural significance of cortical vestibulo-visual integration for visual perception and spatial navigation?
5- How does head-motion signals impact visual computations in the cortex? Is there a computational distinction between passive and active movements and is this consistent with the predictive coding model of cortical function?
6- How does vision impact head-motion coding in the cortex? Is the underlying computation similar for horizontal rotation and linear translation?
7- What is the contribution of otolith vestibular inputs to locomotion speed coding? Do they integrate with motor and optic flow inputs to modulate and shape the cortical speed signal?
8- What is the contribution of angular head velocity cells to cortical function? Do they contribute locally to the generation of the head-direction signal? Do they have unique functions across different cortical networks?

## References

[R1] Agster KL, Burwell RD (2013). Hippocampal and subicular efferents and afferents of the perirhinal, postrhinal, and entorhinal cortices of the rat. Behav Brain Res.

[R2] Alexander AS, Tung JC, Chapman GW, Conner AM, Shelley LE (2022). Adaptive integration of self-motion and goals in posterior parietal cortex. Cell Rep.

[R3] Arenz A, Silver RA, Schaefer AT, Margrie TW (2008). The contribution of single synapses to sensory representation in vivo. Science.

[R4] Arleo A, Dejean C, Allegraud P, Khamassi M, Zugaro MB, Wiener SI (2013). Optic flow stimuli update anterodorsal thalamus head direction neuronal activity in rats. J Neurosci.

[R5] Auger SD, Maguire EA (2013). Assessing the mechanism of response in the retrosplenial cortex of good and poor navigators. Cortex.

[R6] Auger SD, Mullally SL, Maguire EA (2012). Retrosplenial cortex codes for permanent landmarks. PLoS ONE.

[R7] Bassett JP, Taube JS (2001). Neural correlates for angular head velocity in the rat dorsal tegmental nucleus. J Neurosci.

[R8] Bassett JP, Tullman ML, Taube JS (2007). Lesions of the tegmentomammillary circuit in the head direction system disrupt the head direction signal in the anterior thalamus. J Neurosci.

[R9] Baumann O, Mattingley JB (2010). Medial parietal cortex encodes perceived heading direction in humans. J Neurosci.

[R10] Ben-Yishay E, Krivoruchko K, Ron S, Ulanovsky N, Derdikman D, Gutfreund Y (2021). Directional tuning in the hippocampal formation of birds. Curr Biol.

[R11] Bense S, Stephan T, Yousry TA, Brandt T, Dieterich M (2001). Multisensory cortical signal increases and decreases during vestibular galvanic stimulation (fMRI). J Neurophysiol.

[R12] Best C, Lange E, Buchholz HG, Schreckenberger M, Reuss S, Dieterich M (2014). Left hemispheric dominance of vestibular processing indicates lateralization of cortical functions in rats. Brain Struct Funct.

[R13] Bjerknes TL, Langston RF, Kruge IU, Moser EI, Moser MB (2015). Coherence among head direction cells before eye opening in rat pups. Curr Biol.

[R14] Blair HT, Cho J, Sharp PE (1998). Role of the lateral mammillary nucleus in the rat head direction circuit: A combined single unit recording and lesion study. Neuron.

[R15] Blair HT, Sharp PE (1995). Anticipatory head direction signals in anterior thalamus: evidence for a thalamocortical circuit that integrates angular head motion to compute head direction. J Neurosci.

[R16] Bohne P, Schwarz MK, Herlitze S, Mark MD (2019). A new projection from the deep cerebellar nuclei to the hippocampus via the ventrolateral and laterodorsal thalamus in mice. Front Neural Circuits.

[R17] Bouvier G, Sanzeni A, Brunel N, Scanziani M (2022). Inter- and intra-hemispheric sources of vestibular signals to V1.

[R18] Bouvier G, Senzai Y, Scanziani M (2020). Head movements control the activity of primary visual cortex in a luminance-dependent manner. Neuron.

[R19] Bremmer F, Kubischik M, Pekel M, Lappe M, Hoffmann KP (1999). Linear vestibular self-motion signals in monkey medial superior temporal area. Ann N Y Acad Sci.

[R20] Brennan EK, Jedrasiak-Cape I, Kailasa S, Rice SP, Sudhakar SK, Ahmed OJ (2021). Thalamus and claustrum control parallel layer 1 circuits in retrosplenial cortex. Elife.

[R21] Brotchie PR, Andersen RA, Snyder LH, Goodman SJ (1995). Head position signals used by parietal neurons to encode locations of visual stimuli. Nature.

[R22] Brown APY, Cossell L, Strom M, Tyson AL, Velez-Fort M, Margrie TW (2021). Analysis of segmentation ontology reveals the similarities and differences in connectivity onto L2/3 neurons in mouse V1. Sci Rep.

[R23] Bush D, Burgess N (2014). A hybrid oscillatory interference/continuous attractor network model of grid cell firing. J Neurosci.

[R24] Butler JS, Smith ST, Campos JL, Bulthoff HH (2010). Bayesian integration of visual and vestibular signals for heading. J Vis.

[R25] Butler WN, Smith KS, van der Meer MAA, Taube JS (2017). The head-direction signal plays a functional role as a neural compass during navigation. Curr Biol.

[R26] Calton JL, Stackman RW, Goodridge JP, Archey WB, Dudchenko PA, Taube JS (2003). Hippocampal place cell instability after lesions of the head direction cell network. J Neurosci.

[R27] Campagner D, Vale R, Tan YL, Iordanidou P, Arocas OP (2020). A cortico-collicular circuit for accurate orientation to shelter during escape. bioRxiv.

[R28] Carriot J, Jamali M, Chacron MJ, Cullen KE (2017). The statistics of the vestibular input experienced during natural self-motion differ between rodents and primates. J Physiol.

[R29] Carvalho MM, Tanke N, Kropff E, Witter MP, Moser MB, Moser EI (2020). A brainstem locomotor circuit drives the activity of speed cells in the medial entorhinal cortex. Cell Rep.

[R30] Chabrol FP, Arenz A, Wiechert MT, Margrie TW, DiGregorio DA (2015). Synaptic diversity enables temporal coding of coincident multisensory inputs in single neurons. Nat Neurosci.

[R31] Chaplin TA, Margrie TW (2020). Cortical circuits for integration of self-motion and visual-motion signals. Curr Opin Neurobiol.

[R32] Chen A, DeAngelis GC, Angelaki DE (2011). Convergence of vestibular and visual self-motion signals in an area of the posterior sylvian fissure. J Neurosci.

[R33] Chen G, Manson D, Cacucci F, Wills TJ (2016). Absence of visual input results in the disruption of grid cell firing in the mouse. Curr Biol.

[R34] Chen LL, Lin LH, Green EJ, Barnes CA, McNaughton BL (1994). Head-direction cells in the rat posterior cortex - I. anatomical distribution and behavioral modulation. Experimental Brain Research.

[R35] Cho J, Sharp PE (2001). Head direction, place, and movement correlates for cells in the rat retrosplenial cortex. Behav Neurosci.

[R36] Clark BJ, Bassett JP, Wang SS, Taube JS (2010). Impaired head direction cell representation in the anterodorsal thalamus after lesions of the retrosplenial cortex. J Neurosci.

[R37] Crapse TB, Sommer MA (2008). Corollary discharge across the animal kingdom. Nat Rev Neurosci.

[R38] Cullen KE (2019). Vestibular processing during natural self-motion: implications for perception and action. Nat Rev Neurosci.

[R39] Cullen KE, Taube JS (2017). Our sense of direction: progress, controversies and challenges. Nat Neurosci.

[R40] Dannenberg H, Kelley C, Hoyland A, Monaghan CK, Hasselmo ME (2019). The firing rate speed code of entorhinal speed cells differs across behaviorally relevant time scales and does not depend on medial septum inputs. J Neurosci.

[R41] Dannenberg H, Lazaro H, Nambiar P, Hoyland A, Hasselmo ME (2020). Effects of visual inputs on neural dynamics for coding of location and running speed in medial entorhinal cortex. Elife.

[R42] DeAngelis GC, Angelaki DE, Murray MM, Wallace MT (2012). The Neural Bases of Multisensory Processes.

[R43] Denney D, Adorjani C (1972). Orientation specificity of visual cortical neurons after head tilt. Exp Brain Res.

[R44] Dokka K, DeAngelis GC, Angelaki DE (2015a). Multisensory integration of visual and vestibular signals improves heading discrimination in the presence of a moving object. J Neurosci.

[R45] Dokka K, MacNeilage PR, DeAngelis GC, Angelaki DE (2015b). Multisensory self-motion compensation during object trajectory judgments. Cereb Cortex.

[R46] Dudchenko PA, Taube JS (1997). Correlation between head direction cell activity and spatial behavior on a radial arm maze. Behav Neurosci.

[R47] Duffy CJ (1998). MST neurons respond to optic flow and translational movement. J Neurophysiol.

[R48] Dugue GP, Tihy M, Gourevitch B, Lena C (2017). Cerebellar re-encoding of self-generated head movements. Elife.

[R49] Ericsson R, Knight R, Johanson Z (2013). Evolution and development of the vertebrate neck. J Anat.

[R50] Fasold O, von Brevern M, Kuhberg M, Ploner CJ, Villringer A (2002). Human vestibular cortex as identified with caloric stimulation in functional magnetic resonance imaging. Neuroimage.

[R51] Fetsch CR, Pouget A, DeAngelis GC, Angelaki DE (2011). Neural correlates of reliability-based cue weighting during multisensory integration. Nat Neurosci.

[R52] Fetsch CR, Turner AH, DeAngelis GC, Angelaki DE (2009). Dynamic reweighting of visual and vestibular cues during self-motion perception. J Neurosci.

[R53] Fetsch CR, Wang S, Gu Y, Deangelis GC, Angelaki DE (2007). Spatial reference frames of visual, vestibular, and multimodal heading signals in the dorsal subdivision of the medial superior temporal area. J Neurosci.

[R54] Finkelstein A, Derdikman D, Rubin A, Foerster JN, Las L, Ulanovsky N (2015). Three-dimensional head-direction coding in the bat brain. Nature.

[R55] Fischer LF, Soto-Albors RM, Buck F, Harnett MT (2020). Representation of visual landmarks in retrosplenial cortex. eLife.

[R56] Fiser A, Mahringer D, Oyibo HK, Petersen AV, Leinweber M, Keller GB (2016). Experience-dependent spatial expectations in mouse visual cortex. Nat Neurosci.

[R57] Fuhrmann F, Justus D, Sosulina L, Kaneko H, Beutel T (2015). Locomotion, theta oscillations, and the speed-correlated firing of hippocampal neurons are controlled by a medial septal glutamatergic circuit. Neuron.

[R58] Galloni AR, Ye Z, Rancz E (2022). Dendritic domain-specific sampling of long-range axons shapes feedforward and feedback connectivity of l5 neurons. J Neurosci.

[R59] Gerlei K, Passlack J, Hawes I, Vandrey B, Stevens H (2020). Grid cells are modulated by local head direction. Nat Commun.

[R60] Giocomo LM, Moser MB, Moser EI (2011). Computational models of grid cells. Neuron.

[R61] Golob EJ, Stackman RW, Wong AC, Taube JS (2001). On the behavioral significance of head direction cells: neural and behavioral dynamics during spatial memory tasks. Behav Neurosci.

[R62] Goodridge JP, Dudchenko PA, Worboys KA, Golob EJ, Taube JS (1998). Cue control and head direction cells. Behav Neurosci.

[R63] Goodridge JP, Taube JS (1995). Preferential use of the landmark navigational system by head direction cells in rats. Behav Neurosci.

[R64] Goodridge JP, Taube JS (1997). Interaction between the postsubiculum and anterior thalamus in the generation of head direction cell activity. J Neurosci.

[R65] Gorgiladze GI, Smirnov GD (1967). [The effect of vestibular stimulation on the neuronal activity of the visual cortex of the cat brain]. Zh Vyssh Nerv Deiat Im I P Pavlova.

[R66] Green J, Adachi A, Shah KK, Hirokawa JD, Magani PS, Maimon G (2017). A neural circuit architecture for angular integration in Drosophila. Nature.

[R67] Grusser OJ, Grusser-Cornehls U, Saur G (1959). Reaktionen einzelner neurone im optischen cortex der katze nach elektrischer polarisation des labyrinths. Pflugers Arch ges Physiol.

[R68] Grusser OJ, Grusser-Cornehls U (1960). Mikroelektrodenuntersuchungen zur konvergenz vestibulärer und retinaler afferenzen an einzelnen neuronen des optischen cortex der katze. Pflugers Arch ges Physiol.

[R69] Grusser OJ, Grusser-Cornehls U (1972). Interaction of vestibular and visual inputs in the visual system. Prog Brain Res.

[R70] Gu Y, Angelaki DE, Deangelis GC (2008). Neural correlates of multisensory cue integration in macaque MSTd. Nat Neurosci.

[R71] Guitchounts G, Lotter W, Dapello J, Cox D (2022). Stable 3d head direction signals in the primary visual cortex. bioRxiv.

[R72] Guitchounts G, Masis J, Wolff SBE, Cox D (2020). Encoding of 3d head orienting movements in the primary visual cortex. Neuron.

[R73] Harland B, Grieves RM, Bett D, Stentiford R, Wood ER, Dudchenko PA (2017). Lesions of the head direction cell system increase hippocampal place field repetition. Curr Biol.

[R74] Hennestad E, Witoelar A, Chambers AR, Vervaeke K (2021). Mapping vestibular and visual contributions to angular head velocity tuning in the cortex. Cell Rep.

[R75] Hinman JR, Brandon MP, Climer JR, Chapman GW, Hasselmo ME (2016). Multiple running speed signals in medial entorhinal cortex. Neuron.

[R76] Horn G, Hill RM (1969). Modifications of receptive fields of cells in the visual cortex occurring spontaneously and associated with bodily tilt. Nature.

[R77] Horn G, Stechler G, Hill RM (1972). Receptive fields of units in the visual cortex of the cat in the presence and absence of bodily tilt. Exp Brain Res.

[R78] Huang ZJ, Zeng H (2013). Genetic approaches to neural circuits in the mouse. Annu Rev Neurosci.

[R79] Hubel DH, Wiesel TN (1968). Receptive fields and functional architecture of monkey striate cortex. J Physiol.

[R80] Jacob PY, Casali G, Spieser L, Page H, Overington D, Jeffery K (2017). An independent, landmark-dominated head-direction signal in dysgranular retrosplenial cortex. Nature Neuroscience.

[R81] Jacob PY, Poucet B, Liberge M, Save E, Sargolini F (2014). Vestibular control of entorhinal cortex activity in spatial navigation. Front Integr Neurosci.

[R82] Jankowski MM, Ronnqvist KC, Tsanov M, Vann SD, Wright NF (2013). The anterior thalamus provides a subcortical circuit supporting memory and spatial navigation. Front Syst Neurosci.

[R83] Jung R, Kornhuber HH, DF JS (1963). Multisensory convergence on cortical neurons Neuronal Effects of Visual, Acoustic and Vestibular Stimuli in the Superior Convolutions of the Cat’s Cortex. Prog Brain Res.

[R84] Jurgens R, Becker W (2006). Perception of angular displacement without landmarks: evidence for Bayesian fusion of vestibular, optokinetic, podokinesthetic, and cognitive information. Exp Brain Res.

[R85] Justus D, Dalugge D, Bothe S, Fuhrmann F, Hannes C (2017). Glutamatergic synaptic integration of locomotion speed via septoentorhinal projections. Nat Neurosci.

[R86] Keller GB, Bonhoeffer T, Hubener M (2012). Sensorimotor mismatch signals in primary visual cortex of the behaving mouse. Neuron.

[R87] Keller GB, Mrsic-Flogel TD (2018). Predictive processing: a canonical cortical computation. Neuron.

[R88] Keshavarzi S, Bracey EF, Faville RA, Campagner D, Tyson AL (2022). Multisensory coding of angular head velocity in the retrosplenial cortex. Neuron.

[R89] Kim HR, Pitkow X, Angelaki DE, DeAngelis GC (2016). A simple approach to ignoring irrelevant variables by population decoding based on multisensory neurons. J Neurophysiol.

[R90] King WM, Lisberger SG, Fuchs AF (1976). Responses of fibers in medial longitudinal fasciculus (MLF) of alert monkeys during horizontal and vertical conjugate eye movements evoked by vestibular or visual stimuli. J Neurophysiol.

[R91] Klingner CM, Axer H, Brodoehl S, Witte OW (2016). Vertigo and the processing of vestibular information: A review in the context of predictive coding. Neurosci Biobehav Rev.

[R92] Klioutchnikov A, Wallace DJ, Frosz MH, Zeltner R, Sawinski J (2020). Three-photon head-mounted microscope for imaging deep cortical layers in freely moving rats. Nat Methods.

[R93] Klioutchnikov A, Wallace DJ, Sawinski J, Voit KM, Groemping Y, Kerr JND (2022). A three-photon head-mounted microscope for imaging all layers of visual cortex in freely moving mice. Nat Methods.

[R94] Kropff E, Carmichael JE, Moser MB, Moser EI (2015). Speed cells in the medial entorhinal cortex. Nature.

[R95] LaChance PA, Graham J, Shapiro BL, Morris AJ, Taube JS (2022). Landmark-modulated directional coding in postrhinal cortex. Sci Adv.

[R96] Laurens J, Angelaki DE (2018). The brain compass: a perspective on how self-motion updates the head direction cell attractor. Neuron.

[R97] Leinweber M, Ward DR, Sobczak JM, Attinger A, Keller GB (2017). A sensorimotor circuit in mouse cortex for visual flow predictions. Neuron.

[R98] Long X, Deng B, Young CK, Liu GL, Zhong Z (2022). Sharp tuning of head direction and angular head velocity cells in the somatosensory cortex. Adv Sci.

[R99] Lopez C, Blanke O (2011). The thalamocortical vestibular system in animals and humans. Brain Res Rev.

[R100] Magnin M, Jeannerod M, Putkonen P (1974). Vestibular and saccadic influences on dorsal and ventral nuclei of the lateral geniculate body. Exp Brain Res.

[R101] Magnin M, Kennedy H (1979). Anatomical evidence of a third ascending vestibular pathway involving the ventral lateral geniculate nucleus and the intralaminar nuclei of the cat. Brain Res.

[R102] Magnin M, Putkonen PT (1978). A new vestibular thalamic area: electrophysiological study of the thalamic reticular nucleus and of the ventral lateral geniculate complex of the cat. Exp Brain Res.

[R103] Marlinski V, McCrea RA (2008). Activity of ventroposterior thalamus neurons during rotation and translation in the horizontal plane in the alert squirrel monkey. J Neurophysiol.

[R104] Matsuo S, Hosogai M, Nakao S (1994). Ascending projections of posterior canal-activated excitatory and inhibitory secondary vestibular neurons to the mesodiencephalon in cats. Exp Brain Res.

[R105] McFarland WL, Teitelbaum H, Hedges EK (1975). Relationship between hippocampal theta activity and running speed in the rat. J Comp Physiol Psychol.

[R106] McNaughton BL, Barnes CA, O'Keefe J (1983). The contributions of position, direction, and velocity to single unit activity in the hippocampus of freely-moving rats. Exp Brain Res.

[R107] McNaughton BL, Battaglia FP, Jensen O, Moser EI, Moser MB (2006). Path integration and the neural basis of the ‘cognitive map’. Nat Rev Neurosci.

[R108] McNaughton BL, Chen LL, Markus EJ (1991). “Dead reckoning,” landmark learning, and the sense of direction: A neurophysiological and computational hypothesis. Journal of Cognitive Neuroscience.

[R109] Medrea I, Cullen KE (2013). Multisensory integration in early vestibular processing in mice: the encoding of passive vs. active motion. J Neurophysiol.

[R110] Meng H, Angelaki DE (2010). Responses of ventral posterior thalamus neurons to three-dimensional vestibular and optic flow stimulation. J Neurophysiol.

[R111] Meng H, May PJ, Dickman JD, Angelaki DE (2007). Vestibular signals in primate thalamus: properties and origins. J Neurosci.

[R112] Metzler J, Spinelli DN (1979). Tilt-constant receptive fields in the kitten visual cortex. Brain Res.

[R113] Meyer AF, Poort J, O'Keefe J, Sahani M, Linden JF (2018). A head-mounted camera system integrates detailed behavioral monitoring with multichannel electrophysiology in freely moving mice. Neuron.

[R114] Michaiel AM, Abe ET, Niell CM (2020). Dynamics of gaze control during prey capture in freely moving mice. Elife.

[R115] Miura SK, Scanziani M (2022). Distinguishing externally from saccade-induced motion in visual cortex. Nature.

[R116] Muir GM, Brown JE, Carey JP, Hirvonen TP, Della Santina CC (2009). Disruption of the head direction cell signal after occlusion of the semicircular canals in the freely moving chinchilla. J Neurosci.

[R117] Muir GM, Taube JS (2004). Head direction cell activity and behavior in a navigation task requiring a cognitive mapping strategy. Behav Brain Res.

[R118] Nagata S (1986). The vestibulothalamic connections in the rat: a morphological analysis using wheat germ agglutinin-horseradish peroxidase. Brain Res.

[R119] Niell CM, Stryker MP (2010). Modulation of visual responses by behavioral state in mouse visual cortex. Neuron.

[R120] Noel JP, Angelaki DE (2022). Cognitive, systems, and computational neurosciences of the self in motion. Annu Rev Psychol.

[R121] Page HJI, Jeffery KJ (2018). Landmark-based updating of the head direction system by retrosplenial cortex: a computational model. Front Cell Neurosci.

[R122] Papaioannou JN (1973). Effects of caloric labyrinthine stimulation on the spontaneous activity of lateral geniculate nucleus neurons in the cat. Exp Brain Res.

[R123] Parker PRL, Abe ETT, Leonard ESP, Martins DM, Niell CM (2022a). Joint coding of visual input and eye/head position in V1 of freely moving mice. Neuron.

[R124] Parker PRL, Martins DM, Leonard ESP, Casey NM, Sharp SL (2022b). A dynamic sequence of visual processing initiated by gaze shifts. bioRxiv.

[R125] Peck JR, Taube JS (2017). The postrhinal cortex is not necessary for landmark control in rat head direction cells. Hippocampus.

[R126] Perez-Escobar JA, Kornienko O, Latuske P, Kohler L, Allen K (2016). Visual landmarks sharpen grid cell metric and confer context specificity to neurons of the medial entorhinal cortex. Elife.

[R127] Peyrache A, Schieferstein N, Buzsaki G (2017). Transformation of the head-direction signal into a spatial code. Nat Commun.

[R128] Prsa M, Gale S, Blanke O (2012). Self-motion leads to mandatory cue fusion across sensory modalities. J Neurophysiol.

[R129] Rancz EA, Franks KM, Schwarz MK, Pichler B, Schaefer AT, Margrie TW (2011). Transfection via whole-cell recording in vivo: bridging single-cell physiology, genetics and connectomics. Nat Neurosci.

[R130] Rancz EA, Moya J, Drawitsch F, Brichta AM, Canals S, Margrie TW (2015). Widespread vestibular activation of the rodent cortex. J Neurosci.

[R131] Rao RP, Ballard DH (1999). Predictive coding in the visual cortex: a functional interpretation of some extra-classical receptive-field effects. Nat Neurosci.

[R132] Rasmussen RN, Matsumoto A, Arvin S, Yonehara K (2021). Binocular integration of retinal motion information underlies optic flow processing by the cortex. Curr Biol.

[R133] Redish D, Elga AN, Touretzky DS (1996). A coupled attractor model of the rodent head direction system.

[R134] Robertson RG, Rolls ET, Georges-François P, Panzeri S (1999). Head direction cells in the primate pre-subiculum. Hippocampus.

[R135] Roe MB, Aasebo IEJ, Mobarhan M, Lensjo KK, Stober TM (2018). Stable orientation tuning in the freely moving rat: movement-robust orientation selective neurons in the deep layers of the primary visual cortex.

[R136] Rummell BP, Klee JL, Sigurdsson T (2016). Attenuation of responses to self-generated sounds in auditory cortical neurons. J Neurosci.

[R137] Saleem AB, Diamanti EM, Fournier J, Harris KD, Carandini M (2018). Coherent encoding of subjective spatial position in visual cortex and hippocampus. Nature.

[R138] Salinas E, Sejnowski TJ (2001). Gain modulation in the central nervous system: where behavior, neurophysiology, and computation meet. Neuroscientist.

[R139] Sarel A, Finkelstein A, Las L, Ulanovsky N (2017). Vectorial representation of spatial goals in the hippocampus of bats. Science.

[R140] Sargolini F, Fyhn M, Hafting T, McNaughton BL, Witter MP (2006). Conjunctive representation of position, direction, and velocity in entorhinal cortex. Science.

[R141] Sasaki R, Angelaki DE, DeAngelis GC (2017). Dissociation of self-motion and object motion by linear population decoding that approximates marginalization. J Neurosci.

[R142] Sattler NJ, Wehr M (2020). A head-mounted multi-camera system for electrophysiology and behavior in freely-moving mice. Front Neurosci.

[R143] Schneider DM, Nelson A, Mooney R (2014). A synaptic and circuit basis for corollary discharge in the auditory cortex. Nature.

[R144] Seelig JD, Jayaraman V (2015). Neural dynamics for landmark orientation and angular path integration. Nature.

[R145] Sharp PE, Tinkelman A, Cho J (2001). Angular velocity and head direction signals recorded from the dorsal tegmental nucleus of gudden in the rat: implications for path integration in the head direction cell circuit. Behav Neurosci.

[R146] Sharp PE, Turner-Williams S (2005). Movement-related correlates of single-cell activity in the medial mammillary nucleus of the rat during a pellet-chasing task. J Neurophysiol.

[R147] Shinder ME, Taube JS (2011). Active and passive movement are encoded equally by head direction cells in the anterodorsal thalamus. J Neurophysiol.

[R148] Shine JP, Valdes-Herrera JP, Hegarty M, Wolbers T (2016). The human retrosplenial cortex and thalamus code head direction in a global reference frame. J Neurosci.

[R149] Shiroyama T, Kayahara T, Yasui Y, Nomura J, Nakano K (1999). Projections of the vestibular nuclei to the thalamus in the rat: a phaseolus vulgaris leucoagglutinin study. J Comp Neurol.

[R150] Sit KK, Goard MJ (2022). Coregistration of heading to visual cues in retrosplenial cortex. bioRxiv.

[R151] Skaggs WE, Knierim JJ, Kudrimoti HS, McNaughton BL (1995). A model of the neural basis of the rat’s sense of direction. Adv Neural Inf Process Syst.

[R152] Spalla D, Treves A, Boccara CN (2022). Angular and linear speed cells in the parahippocampal circuits. Nat Commun.

[R153] Spiegel EA, Egyed J, Szekely EG (1968). Cortical responses to rotation. II. Responses recorded at the onset of rotation from the second somatic sensory and posterior areas. Acta Otolaryngol.

[R154] Spratling MW (2010). Predictive coding as a model of response properties in cortical area V1. J Neurosci.

[R155] Stackman RW, Golob EJ, Bassett JP, Taube JS (2003). Passive transport disrupts directional path integration by rat head direction cells. J Neurophysiol.

[R156] Stackman RW, Taube JS (1997). Firing properties of head direction cells in the rat anterior thalamic nucleus: dependence on vestibular input. J Neurosci.

[R157] Stahl JS (2004). Using eye movements to assess brain function in mice. Vision Res.

[R158] Sugar J, Witter MP, van Strien NM, Cappaert NL (2011). The retrosplenial cortex: intrinsic connectivity and connections with the (para)hippocampal region in the rat. An interactive connectome. Front Neuroinform.

[R159] Taube JS (1995). Head direction cells recorded in the anterior thalamic nuclei of freely moving rats. J Neurosci.

[R160] Taube JS (2007). The head direction signal: origins and sensory-motor integration. Ann Rev Neurosci.

[R161] Taube JS, Muller RU, Ranck JB (1990a). Head-direction cells recorded from the postsubiculum in freely moving rats. I. Description and quantitative analysis. J Neurosci.

[R162] Taube JS, Muller RU, Ranck JB (1990b). Head-direction cells recorded from the postsubiculum in freely moving rats. II. Effects of environmental manipulations. J Neurosci.

[R163] Thier P, Erickson RG (1992). Responses of visual-tracking neurons from cortical area mst-i to visual, eye and head motion. Eur J Neurosci.

[R164] Tiecks FP, Planck J, Haberl RL, Brandt T (1996). Reduction in posterior cerebral artery blood flow velocity during caloric vestibular stimulation. J Cereb Blood Flow Metab.

[R165] Tomko DL, Barbaro NM, Ali FN (1981). Effect of body tilt on receptive field orientation of simple visual cortical neurons in unanesthetized cats. Exp Brain Res.

[R166] Toyama K, Komatsu Y, Shibuki K (1984). Integration of retinal and motor signals of eye movements in striate cortex cells of the alert cat. J Neurophysiol.

[R167] Turner-Evans D, Wegener S, Rouault H, Franconville R, Wolff T (2017). Angular velocity integration in a fly heading circuit. Elife.

[R168] Valerio S, Taube JS (2012). Path integration: how the head direction signal maintains and corrects spatial orientation. Nat Neurosci.

[R169] Valerio S, Taube JS (2016). Head direction cell activity is absent in mice without the horizontal semicircular canals. J Neurosci.

[R170] van der Meer MAA, Richmond Z, Braga RM, Wood ER, Dudchenko PA (2010). Evidence for the use of an internal sense of direction in homing. Behav Neurosci.

[R171] Vanni-Mercier G, Magnin M (1982). Single neuron activity related to natural vestibular stimulation in the cat’s visual cortex. Exp Brain Res.

[R172] Velez-Fort M, Bracey EF, Keshavarzi S, Rousseau CV, Cossell L (2018). A circuit for integration of head- and visual-motion signals in layer 6 of mouse primary visual cortex. Neuron.

[R173] Velez-Fort M, Rousseau CV, Niedworok CJ, Wickersham IR, Rancz EA (2014). The stimulus selectivity and connectivity of layer six principal cells reveals cortical microcircuits underlying visual processing. Neuron.

[R174] Vinepinsky E, Cohen L, Perchik S, Ben-Shahar O, Donchin O, Segev R (2020). Representation of edges, head direction, and swimming kinematics in the brain of freely-navigating fish. Sci Rep.

[R175] Voigts J, Siegle JH, Pritchett DL, Moore CI (2013). The flexDrive: an ultra-light implant for optical control and highly parallel chronic recording of neuronal ensembles in freely moving mice. Front Syst Neurosci.

[R176] von Holst E, Mittelstaedt H (1950). Das Reafferenzprinzip. Wechselwirkungen zwischen Zentralnervensystem und Peripherie Naturwissenschaften.

[R177] Wallace DJ, Greenberg DS, Sawinski J, Rulla S, Notaro G, Kerr JN (2013). Rats maintain an overhead binocular field at the expense of constant fusion. Nature.

[R178] Wenzel R, Bartenstein P, Dieterich M, Danek A, Weindl A (1996). Deactivation of human visual cortex during involuntary ocular oscillations. A PET activation study. Brain.

[R179] Whitlock JR, Pfuhl G, Dagslott N, Moser MB, Moser EI (2012). Functional split between parietal and entorhinal cortices in the rat. Neuron.

[R180] Wiener SI, Paul CA, Eichenbaum H (1989). Spatial and behavioral correlates of hippocampal neuronal activity. J Neurosci.

[R181] Winter SS, Clark BJ, Taube JS (2015a). Spatial navigation. Disruption of the head direction cell network impairs the parahippocampal grid cell signal. Science.

[R182] Winter SS, Mehlman ML, Clark BJ, Taube JS (2015b). Passive transport disrupts grid signals in the parahippocampal cortex. Curr Biol.

[R183] Wyss JM, Van Groen T (1992). Connections between the retrosplenial cortex and the hippocampal formation in the rat: a review. Hippocampus.

[R184] Yoder RM, Clark BJ, Brown JE, Lamia MV, Valerio S (2011). Both visual and idiothetic cues contribute to head direction cell stability during navigation along complex routes. J Neurophysiol.

[R185] Yoder RM, Peck JR, Taube JS (2015). Visual landmark information gains control of the head direction signal at the lateral mammillary nuclei. J Neurosci.

[R186] Zhang K (1996). Representation of spatial orientation by the intrinsic dynamics of the head-direction cell ensemble: a theory. J Neurosci.

[R187] Zong W, Obenhaus HA, Skytoen ER, Eneqvist H, de Jong NL (2022). Large-scale two-photon calcium imaging in freely moving mice. Cell.

[R188] Zong W, Wu R, Li M, Hu Y, Li Y (2017). Fast high-resolution miniature two-photon microscopy for brain imaging in freely behaving mice. Nat Methods.

